# Clinical patient registry recruitment and retention: a survey of patients in two chronic disease registries

**DOI:** 10.1186/s12874-017-0343-3

**Published:** 2017-04-17

**Authors:** Daniel H. Solomon, Nancy A. Shadick, Michael E. Weinblatt, Michelle Frits, Christine Iannaccone, Agnes Zak, Joshua R. Korzenik

**Affiliations:** 1 Division of Rheumatology, Brigham and Women’s Hospital, 75 Francis Street, Boston, MA 02115 USA; 2 Division of Pharmacoepidemiology, Brigham and Women’s Hospital, Boston, MA USA; 3 Division of Gastroenterology, Brigham and Women’s Hospital, Boston, MA USA

**Keywords:** Rheumatoid arthritis, Inflammatory bowel disease, Patient survey, Patient registry

## Abstract

**Background:**

The collection of routine clinical data in the setting of research registries can serve an important role in understanding real world care. However, relatively little is known about the patient experience in registries, motivating us to survey patients enrolled in two chronic disease registries.

**Methods:**

We conducted similar surveys in two disease-based registries based at one academic medical center in the US. One group of patients with rheumatoid arthritis (RA) had been enrolled in a registry, and we focused on retention factors. In a second group of patients with inflammatory bowel disease (IBD) recently enrolled or considering enrollment, we examined factors that would influence their enrollment and willingness to answer frequent questionnaires and give biospecimens. The surveys were analyzed using descriptive statistics and the two cohorts were compared using nonparametric and chi-square tests.

**Results:**

We received 150 (50%) completed surveys from RA and 169 (63%) from IBD patients. Mean age of subjects was 62 years in RA and 43 in IBD with more women respondents with RA (83%) than IBD (62%). The two groups described very similar factors as the top three motivations for participation: desire to help others, desire to improve care of own disease, and ease of volunteering. Preferred methods of surveying included mail, e-mail, but telephone was not favored; age was an important correlate of this preference. Respondents preferred surveys either every 1–3 months (28.7% RA and 55.0% IBD) or every 4–6 months (50.7% RA and 29.0% IBD). They differed in the preference for payment for answering surveys with 68.0% with RA answering that no payment was necessary but only 36.1% with IBD felt similarly.

**Conclusions:**

Patients engaged in clinical registries demonstrate a high level of commitment to improve care and many report a willingness to answer questions relatively frequently.

**Electronic supplementary material:**

The online version of this article (doi:10.1186/s12874-017-0343-3) contains supplementary material, which is available to authorized users.

## Background

Patient registries facilitate data collection during routine clinical care, allowing for observational studies of a disease and/or treatment over time [1]. Registries have grown more numerous and continue to expand [2]. Questionnaire items from registries often focus on disease and treatment outcomes, treatment decision-making, quality of life, and the safety and effectiveness of therapies [1]; most of these items utilize patient reported outcomes (PROs). Patient registries are employed to study chronic conditions, such as diabetes [3], cancer [4], IBD [5], and musculoskeletal disorders, such as rheumatoid arthritis (RA) [6–8]. The design of patient registries depends on the specific questions of interest, but the frequency of patient questionnaires, visit, and biospecimen collections is always an area of concern. Moreover, recruitment and retention of patients presents ongoing challenges to investigators.

We have previously conducted several focus groups with patients involved in chronic disease registries or others uninvolved but with chronic diseases [9]. The focus groups uncovered a variety of issues for patient engagement in registries, including participant burden, methods for survey completion, and content area. To further examine these issues, we conducted parallel surveys among two chronic disease cohorts – one with RA and another with inflammatory bowel disease (IBD); both were enrolled or considering enrollment in patient registries.

## Methods

### Study participants and recruitment

We conducted a one-time survey of patients with RA who currently participate in a single center registry (BRASS), as well as a one-time survey of patients with IBD who have recently enrolled or considering enrollment in a single center registry (BrITR). Both registries are conducted at a large academic medical center in Boston Massachusetts. The RA registry involves over 30 providers and the IBD registry involves 8 providers. The RA registry has been operational for 14 years and the IBD registry for 4. Both have been funded through a variety of pharmaceutical sponsors, foundations, and federal grants. The design and methods for BRASS have been well described [6]. Briefly, BRASS has enrolled 1460 patients diagnosed by their rheumatologist with RA. As part of BRASS, they fill in written questionnaires twice per year at 6-month intervals. They are mailed to participants once per year, and the other is given to the patients at an in-person visit. During the in-person visit, a research assistant also interviews the patient to gather additional data. Subjects are asked to provide blood once per year and have hand x-rays performed every 2–3 years. The BrITR registry has enrolled 957 patients diagnosed with IBD based on a diagnosis of Crohn’s disease or ulcerative colitis. Participants answer questionnaires at baseline through paper surveys and give blood/stool and biopsies at colonoscopy or follow up depending on a change in clinical status.

We recruited subjects from each cohort with slightly different methods. The BRASS participants were mailed questionnaires by US mail, since many have not provided the investigators with e-mail addresses. Subjects were sent the questionnaire and a cover letter explaining the goals of the research. They were also offered an honorarium (gift card) for successful completion of the survey. The subjects in BrITR were recruited via the same cover letter, but it was sent by e-mail. An identical honorarium was offered. A follow-up reminder invitation was re-sent to non-respondents 3 weeks after the initiation mailing.

### Survey items

The surveys covered several areas in common (see Additional file 1 for surveys). These include: sociodemographics, quality of life, [10, 11] reasons for enrollment, reasons to stay enrolled, methods for survey completion, frequency of surveys, honorarium amounts, survey topics, and specific areas that should be covered in questionnaires. The survey directed at patients with RA also asked about reasons for missing questionnaires, while the survey directed at patients with IBD included questions about biospecimen collection.

Many of the questions asked respondents to rank the top three reasons for enrollment or ongoing participation in the registry. To allow for easier comparison across items and across registries, we created a score from 0 to 3, where 0 signified no ratings of an item in the top 3 and 3 signified the top rated item across all respondents.

### Statistical analysis

Descriptive analyses were used to assess patient characteristics and their survey responses, including proportions and means. Without specific a priori hypotheses, we also compared responses across the two groups, using Chi-Square tests or Kruskal-Wallis tests. The goal of the comparison between the two registries was to determine if different patients had different attitudes regarding participation. The univariate correlation between selected variables was assessed.

We aimed to recruit at least 150 subjects from each cohort and achieve ≥50% recruitment of those invited. After this was achieved, no further recruitment occurred. All analyses were conducted using SAS (Version 9.4, Cary NC).

## Results

We recruited 150 (50%) patients with RA from BRASS and 169 (63%) patients with IBD from BrITR. Their characteristics are described in Table 1. There were more women respondents in BRASS (83%) than BrITR (62%) and BRASS respondents were on average older (62 years) than BrITR (43 years). Both cohorts were primarily white and non-hispanic (94–95%) and most had attended some or graduated college (85–90%). The average quality of life ratings for both cohorts were moderate to good.

**Table 1:** Baseline characteristics of survey participants

	BRASS (RA)	BrITR (IBD)
	n (%) or mean ± SD	
Total surveys returned	150 (50.0)^a^	169 (63.1)^a^
Sex, female	124 (82.7)	103 (62.0)
Age, years	61.7 ± 12.2	42.9 ± 13.5
< 45	14 (9.3)	103 (62.1)
45–64	60 (40.0)	44 (26.5)
65+	76 (50.7)	19 (11.4)
Race/ethnicity		
White/non-Hispanic	141 (94)	157 (94.6)
White/Hispanic	3 (2.0)	2 (1.2)
Black/non-Hispanic	3 (2.0)	7 (4.2)
Asian	3 (2.0)	1 (0.6)
Other	3 (2.0)	0 (0.0)
Highest level of education		
High school or below	16 (10.7)	10 (6.0)
Some college	16 (10.7)	19 (11.4)
College graduate	62 (41.3)	57 (34.3)
Beyond college	56 (37.3)	80 (48.2)
SIBDQ^b^	…	5.3 ± 1.0
EuroQol^b^	0.8 ± 0.2	…

^a^This percentage refers to the percent of all patients invited from a given registry. ^b^The Short Inflammatory Bowel Disease Questionnaire is a health-related quality of life tool measuring physical, emotional, and social status. Scoring is 1 (poor) to 7 (excellent) health-related quality of life. The EuroQol (EQ-5D Index) is a standardized instrument used to measure quality of life in five dimensions that include mobility, self-care, usual activities, pain/discomfort, and anxiety/depression. Scoring is on a 0 to 1 scale, with 0 being death and 1 being perfect health. The ellipsis (…) was used when the responses used among the different cohorts were not identical

The same three reasons were most frequently selected for motivating factors for participation (see Table 2): desire to help others, hope that it would help manage RA or IBD, and ease of volunteering. The next most common response was that the physician had convinced the patient to participate. Honorarium and gifts were not felt to be important. Most respondents stated that they would stay involved in the registry no matter what. Specific factors associated with remaining involved included getting general information on a patient’s condition, getting feedback about surveys responses, and getting paid to answer surveys.

**Table 2:** Motivations for participation and preferences for survey completion

	BRASS (RA) mean ± SD	BrITR (IBD) mean ± SD	*p*-value
What motivated you to participate in BRASS/BrITR? Please rank the top 3^a^			
My doctor convinced me to	0.8 ± 1.1	0.7 ± 1	0.66
A family member or friend convinced me	0.0 ± 0.1	0.1 ± 0.4	0.01
My desire to help others	2.3 ± 1	2.4 ± 0.9	0.47
I hoped it would help me take care of my RA/IBD	1.0 ± 1.1	1.2 ± 1.2	0.21
It was easy to volunteer	0.9 ± 1	1.0 ± 0.9	0.03
I like the gifts (pens, magnets, parking, etc.)	0.0 ± 0.3	0.1 ± 0.4	0.08
The research assistants are nice	0.1 ± 0.4	0.2 ± 0.5	0.41
What would increase your willingness to remain in BRASS/BrITR? Please rank the top 3^a^			
Free parking	0.7 ± 1.0	0.4 ± 0.8	0.009
Fewer questionnaires	0.4 ± 0.9	0.3 ± 0.8	0.65
Getting paid to answer surveys	0.4 ± 0.8	1.0 ± 1.2	<0.001
Getting feedback on my answers	0.9 ± 1.2	1.0 ± 1.2	0.28
Getting general information on my condition	0.9 ± 1.0	1.0 ± 1.2	0.71
I will stay enrolled no matter what	1.4 ± 1.3	1.3 ± 1.3	0.64
What are your preferred methods of completing surveys? Please rank the top 3^a^			
Paper surveys mailed home	2.1 ± 1.2	0.3 ± 0.6	<0.001
Paper or tablet surveys at clinic	0.9 ± 1.1	0.6 ± 0.9	0.001
Phone survey	0.2 ± 0.5	0.1 ± 0.4	0.23
E-mail survey	1.1 ± 1.1	2.1 ± 1.0	<0.001
Internet survey	0.8 ± 1.1	1.9 ± 1.1	<0.001
Survey using a PDA (application)	0 .2 ± 0.5	0.9 ± 1.0	<0.001

^a^To allow for easier comparison across items and across registries, we created a score from 0 to 3, where 0 signified no ratings of an item in the top 3, and 3 signified the top rated item across all respondents. The ratings were 3 = most important, 2 = second most important, and 1 = third most important. For items not rated in the top 3, zero was assigned. *Abbreviations: RA* rheumatoid arthritis, *IBD* inflammatory bowel disease. *P*-values calculated using Kruskal-Wallis tests

The preferred methods for completing surveys differed across cohorts (see Table 2). In BRASS, a mailed paper survey was the most preferred method. This was followed by e-mail, in clinic, and internet surveys. Telephone and a PDA application survey were least preferred. Unlike BRASS, the BrITR cohort did not prefer mailed paper surveys, but they did prefer a PDA application survey. Similar to BRASS, they also preferred e-mail and internet surveys. We grouped both cohorts and examined the unadjusted correlation between age categories (<45 years, 45–64 years, and 65+ years) and survey preferences (see Table 3). Younger participants preferred surveys via e-mail, internet or phone applications (all *p* < 0.001). We also examined the unadjusted correlation between sex and survey preferences and found some statistically significant differences such as males were more likely to prefer an internet survey than females (see Additional file 2).

**Table 3:** Preferred methods of completing survey by age category combining both cohorts

	Age (years)			
	<45 years	45-64 years	65+ years	*p*-value
Paper surveys mailed home	0.4 ± 0.7	1.1 ± 1.2	2.1 ± 1.2	<0.001
Paper or tablet at clinic	0.6 ± 1.1	0.7 ± 1.0	0.9 ± 1.0	0.12
Phone survey	0.1 ± 0.4	0.1 ± 0.4	0.2 ± 0.6	0.03
E-mail survey	2.0 ± 1.0	1.8 ± 1.2	1.1 ± 1.2	<0.001
Internet survey	1.9 ± 1.1	1.4 ± 1.2	0.6 ± 1.0	<0.001
Survey using a PDA (application)	0.9 ± 1.0	0.4 ± 0.8	0.2 ± 0.5	<0.001

To allow for easier comparison across items and across registries, we created a score from 0 to 3, where 0 signified no ratings of an item in the top 3, and 3 signified the top rated item across all respondents. The ratings were 3 = most important, 2 = second most important, and 1 = third most important. For items not rated in the top 3, zero was assigned. *P*-values calculated using Kruskal-Wallis tests

Participants preferences for survey intervals differed across registries (see Table 4). The majority (51%) of BRASS participants stated that every 4–6 months was the most frequent they would answer surveys, while 55% of BrITR participants were willing to answer surveys every 1–3 months (*P* < 0.001). There were also differences in the preferences for honorarium. While the majority (68%) of subjects in BRASS did not consider an honorarium motivating, far fewer (36%) respondents from BrITR said no honorarium was necessary (*p* < 0.001).

**Table 4:** Questionnaire frequency and motivating honorarium

	BRASS (RA)	BrITR (IBD)	*p*-value
At most, how often would you be willing to respond to questionnaires or surveys?			
Every 1–3 months	43 (28.7)	93 (55.0)	<0.001
Every 4–6 months	76 (50.7)	49 (29.0)	
Every 7–12 months	29 (19.3)	19 (11.2)	
Every 13–24 months	1 (0.7)	6 (3.6)	
If BRASS/BrITR offered to pay participants to answer surveys, what would be a motivating payment for you?			
No payment necessary	102 (68.0)	61 (36.1)	<0.001
$10	14 (9.3)	24 (14.2)	
$20	10 (6.7)	58 (34.3)	
$25	23 (15.3)	…	
> $30	…	24 (14.2)	

*P*-value from Chi-Square test. The ellipsis (…) was used when the responses used among the different cohorts were not identical. Percentages may not add to 100% because of rounding

We also oversampled BRASS participants who had been inconsistent responders to learn why they missed surveys (see Table 5). The most common reason given was that they had changed rheumatology providers away from the hospital running BRASS. Others answered that life events had interrupted their participation.

**Table 5:** BRASS specific questions among subset of 52 participants who had been recently inactive

	mean ± SD
What is the reason for your missed visits? Please rank the top 3^a^	
Too much blood work	0.1 ± 0.5
Too many x-rays	0.2 ± 0.7
Too time consuming	0.4 ± 0.8
No longer see rheumatologist at BRASS institution	0.9 ± 1.4
Surveys are too long	0.3 ± 0.8
Other life events conflict	0.7 ± 1.1

^a^To allow for easier comparison across items and across registries, we created a score from 0 to 3, where 0 signified no ratings of an item in the top 3, and 3 signified the top rated item across all respondents. The ratings were 3 = most important, 2 = second most important, and 1 = third most important. For items not rated in the top 3, zero was assigned. Recently inactive was defined as missing 2 consecutive annual visits without dropping out

The BrITR registry has a strong interest in frequent specimen collection and a series of questions were designed to determine willingness for different frequencies (see Table 6). Most (81%) participants reported that intestinal biopsies could be taken at every endoscopy. However, participants were much less willing to provide stool specimens with the majority answering every 3–12 months as a preferred time period, but 15% said never. Participants reported similar interval preferences for urine specimens.

**Table 6:** BrITR specific questions regarding biospecimen collection

	N (%)
At most, how often would you be willing to provide a biopsy of intestine from endoscopy?	
Every time I have endoscopy	134 (80.7)
Every other time I have endoscopy	25 (15.1)
Never	7 (4.2)
At most, how often would you be willing to provide a stool specimen?	
Every 2 weeks	7 (4.2)
Every 4–6 weeks	20 (12.0)
Every 2 months	6 (3.6)
Every 3 months	23 (13.9)
Every 6 months	33 (19.9)
Every 12 months	53 (31.9)
Never	24 (14.5)
At most, how often would you be willing to provide a urine specimen?	
Every 2 weeks	11 (6.6)
Every 4–6 weeks	34 (20.5)
Every 2 months	9 (5.4)
Every 3 months	38 (22.9)
Every 6 months	38 (22.9)
Every 12 months	33 (19.9)
Never	3 (1.8)

## Discussion

Patient registries provide important clinical research data for many chronic conditions. They allow improved reporting of PROs and enhance patient engagement in the research process. However, little is known about the patient perspective on registries. As a follow-up to a prior set of focus groups, [9] we surveyed two cohorts of patients who participate in chronic disease registries. There were important similarities across the cohorts regarding motivating factors, but also important differences regarding preferences for how to receive and answer questionnaires, frequency of questionnaires, and honorarium. The differences in the questionnaire administration appeared to correlate with age and gender. The comparisons across registry cohorts were made without respect to a priori hypotheses. However, by asking many of the same questions, we were able to assess whether the factors associated with registry participation were similar or differed and to determine if factors such as age and gender were correlates.

Several implications of this research may impact the design and conduct of patient registries. First, successful recruitment of patients likely depends on appealing to patient’s sense of altruism; most respondents note that they participate in a registry based on a desire to help others. They also want the experience to provide potential benefits to their own care, and thus it might be worthwhile to demonstrate this possibility to patients during recruitment. This might include graphical representation of their symptoms over time and in comparison to the totality of participants.

Second, it should be easy to participate; thus, investigators should consider flexible survey modalities and intervals. While there may be some age-related preferences for how to complete surveys, different participants prefer different methods. This suggests that registries should consider a tailored approach to provide questionnaires in the manner preferred by patients, even if they must provide them in different manners for different participants. In keeping with this theme of tailoring, some subjects will agree to a more frequent schedule of questionnaires. It may be possible to follow a subset of patients more frequently. Finally, some honorarium is appreciated by many subjects, but not all. We did offer a gift card for participation in this survey as this is recommended by our Human Ethics Board for studies with minimal benefits accruing to the subject.

Those who had missed in-person visits for BRASS typically reported that they were no longer seeing a rheumatologist at the sponsoring hospital or that they had life events interrupt their participation. This speaks to the importance of allowing subjects to participate without face to face meetings for those who change their place of care. In 2012, BRASS began consenting patient to remain enrolled in the study as ‘electronic medical record’ only participants. These patients no longer answer questionnaires or have in-person visits, but allow the study to collect data from their medical record.

While this survey provides new information regarding patient preferences with respect to clinical registries, we surveyed participants and not those who had refused participation. Thus, we are not obtaining a complete understanding of the issues. Further, both cohorts were from the same institution and most were White non-Hispanic. Our prior patient focus group work highlighted the fact that Hispanic patients demonstrated a poor understanding of patient registries. Testing successful strategies for recruiting minorities into patient registries is important future work. Our prior work also demonstrated that engaging patients in deciding on the research agenda enhances initial participation and retention in registries; we did not pursue this issue in the current survey. Some correlates of registry participation may be similar to factors affecting participation in patient surveys.

## Conclusion

In conclusion, we collected survey data regarding patient participation in clinical registries. These data should help inform registry design and recruitment/retention strategies. With the likely continued growth in patient registries collecting PROs, patients must continue to be engaged as active participants. As well, better methods for data collection should continue to enhance registry research.

## Additional files

Additional file 1: These files contain the surveys used to conduct the research described in the manuscript. (ZIP 388 kb)

Additional file 2: This table describes the preferred methods of completing survey by gender after combining both cohorts. (DOCX 11 kb)

## Abbreviations

BRASS: Brigham Rheumatoid Arthritis Sequential Study
BrITR: Brigham InflammaTory Bowel Disease Registry
IBD: Inflammatory bowel disease
PDA: Personal Digital Assistant
PRO: Patient Reported Outcome
RA: Rheumatoid arthritis
