# Supplementary material for: Clinical patient registry recruitment and retention: a survey of patients in two chronic disease registries
Source: BMC Med Res Methodol. 2017 Apr 17;17:59. doi: 10.1186/s12874-017-0343-3 (PMC5392954; doi:10.1186/s12874-017-0343-3)
Supplement: Supplementary file 1 — These files contain the surveys used to conduct the research described in the manuscript. (ZIP 388 kb) [file 12874_2017_343_MOESM1_ESM.zip › Survey.BRASS.final.solomonR3.pdf]

This survey is designed to collect information related to how you feel about participating in a research registry like BRASS. Please answer each question to the best of your ability.

1) What motivated you to participate in BRASS? **Rank** the top 3, marking the most important with the number 1, the second most important with the number 2, and the third most important with the number 3.

____ My doctor convinced me
___ A family member or friend convinced me
____ My desire to help others
____ I hoped it would help me take better care of my RA
____ It was easy to volunteer
____ I like the gifts (pens, magnets, parking, etc.)
____ The research assistants are nice
____ Other: ____

2) What would increase your willingness to stay involved or become more involved in BRASS? **Rank** the top 3, marking the most important with the number 1, the second most important with the number 2, and the third most important with the number 3.

____ Continue offering free parking
____ Fewer questionnaires
____ Getting paid to answer surveys
____ Getting feedback from the registry on my survey answers
____ Getting more general information from the registry about RA
____ Having educational presentations from the registry doctors about RA
____ Having a chance to talk with other patients in the registry
____ Less blood work
____ Less x-rays
____Less in-person study visits
____Being able to schedule separate appointment for BRASS
____ I will stay enrolled and answer all questionnaires no matter what

The next few questions will ask you specifically about BRASS surveys and questionnaires.

3) What would be your preferred method of filling out surveys? **Rank** the top 3, marking the most preferred with the number 1, the second most preferred with the number 2, and the third most preferred with the number 3.

____ Paper survey at home that I mail back
____ Paper survey at home that I can bring to the clinic
____ Paper survey at the clinic
____ Tablet/computer survey at the clinic
____ Phone survey
____ Email survey (secure and encrypted)
____ Survey on the internet/web (secure and encrypted)
____ Survey on a smart phone application (secure and encrypted)
____ Other: ____

4) At most, how often would you be willing to respond to mailed BRASS surveys? Pick **one** answer.

- ☐ Every 1-3 months
- ☐ Every 4-6 months
- ☐ Every 7-12 months
- ☐ Every 13-24 months

5) Would you consider answering BRASS surveys more often if they were shorter? For example, if BRASS wanted you to report on specific RA symptoms, like pain and swelling, with a 1-2 minute checklist. Would you consider answering such questions, and how often? Pick **one** answer.

- ☐ Would not answer
- ☐ Yes, about every 1 week
- ☐ Yes, about every 2 weeks
- ☐ Yes, about every 1 month
- ☐ Yes, about every 2 months

6) If BRASS offered to pay participants to answer surveys what would be a motivating payment for you? Pick **one** answer.

- ☐ No payment necessary
- ☐ $10
- ☐ $20
- ☐ $25

7) What topics should BRASS ask you about on surveys? **Rank** the top 5, marking the most important with the number 1, the second most important with the number 2, and so on until you get to the number 5.

____ Medications for RA
____ Medications, other than for RA
____ Emotional health
____ Coping strategies (such as meditation or physical activity)
____ RA disease activity
____ RA disease complications (such as heart problems or nerve damage)
____ Economic effects of RA
____ Diet and nutrition
____ Alternative therapy (such as supplements and health food preparations)
____ Other medical conditions
____ Other: ____

8) Is there anything about the current BRASS surveys that you think should be changed? Check **all** that apply.

- ☐ Make it shorter
- ☐ Make it longer
- ☐ Larger size text (larger font)
- ☐ Put more space between the questions (text is too crowded)
- ☐ Put all answer choices going down the page instead of across (vertically instead of horizontally)
- ☐ I would not change anything
- ☐ Other: ____

9) If you have ever left questions blank on a BRASS survey, what were your reasons for doing so? Check **all** that apply.

☐ Question is too personal in nature
☐ I did not understand the question
☐ I did not think it applied to me
☐ None of the given choices matched my answer
☐ I did not know or could not remember the answer
☐ Question did not apply to my RA, but to different health issue
☐ Ran out of steam (survey too long)
☐ I do not leave questions blank
☐ Other: ____

The next few questions will ask you specifically about BRASS in-person study visits (not mailed surveys).

10) At most, how often would you be willing to complete an in-person BRASS study visit? Pick **one** answer.

☐ Every 3 months
☐ Every 6 months
☐ Every 12 months
☐ Every 18 months

11) What is the maximum length of time you would be willing to spend at an in-person BRASS study visit? Pick **one** answer.

☐ No more than 15 minutes
☐ No more than 30 minutes
☐ No more than 45 minutes
☐ No more than 1 hour

12) If BRASS offered to pay participants to complete in-person study visits, what would be a motivating payment for you? Pick **one** answer.

☐ No payment necessary
☐ $10
☐ $20
☐ >$30

13) When would it be convenient for you to complete an in-person BRASS study visit? **Rank** the top 3, marking the most convenient with the number 1, the second most convenient with the number 2, and the third most convenient with the number 3.

____ At my regularly scheduled rheumatology appointment
____ At any appointment I have scheduled in the main hospital (75 Francis Street)
____ At any appointment I have scheduled in any location (such as 850 Boylston St. or Foxborough)
____ Set up a separate time to come in to the hospital for a research study visit
____ Other________________________________

14) What would be your preferred method of contact from study staff regarding your survey answers or study visits? **Rank** the top 3, marking the most preferred with the number 1, the second most preferred with the number 2, and the third most preferred with the number 3.

___ Mail
___ Phone
___ Email
___ Text message
___ Do not contact me with questions about my survey answers or study visits

15) Please check the statement that is most accurate regarding the number of research studies you are asked to participate in. Pick **one** answer.

☐ The number of research studies I am asked to participate in is appropriate
☐ The number of research studies I am asked to participate in is too few
☐ The number of research studies I am asked to participate in is too many

Thank you for completing this survey!
