# Supplementary material for: Clinical patient registry recruitment and retention: a survey of patients in two chronic disease registries
Source: BMC Med Res Methodol. 2017 Apr 17;17:59. doi: 10.1186/s12874-017-0343-3 (PMC5392954; doi:10.1186/s12874-017-0343-3)
Supplement: Supplementary file 1 — These files contain the surveys used to conduct the research described in the manuscript. (ZIP 388 kb) [file 12874_2017_343_MOESM1_ESM.zip › Survey.BrITR.final.solomonR3.pdf]

This survey is designed to collect information related to how you feel about participating in a research registry like BrITR. Please answer each question to the best of your ability.

1) What motivated you to participate in the BrITR study (BWH IBD Registry)? Rank the top 3, marking the most important with the number 1, the second most important with the number 2, and the third most important with the number 3.
   ___ My doctor convinced me
   ___ A family member or friend convinced me
   ___ My desire to help others
   ___ I hoped it would help me take better care of my IBD
   ___ It was easy to volunteer
   ___ I like the gifts (pens, magnets, parking, etc.)
   ___ The research assistants are nice
   ___ Other: ____

2) What would increase your willingness to stay involved in the registry? Rank the top 3, marking the most important with the number 1, the second most important with the number 2, and the third most important with the number 3.
   ___ Free parking
   ___ Fewer surveys
   ___ Getting paid to answer surveys
   ___ Getting feedback from the registry on my survey answers
   ___ Getting more general information from the registry about IBD
   ___ Having educational presentations from the registry providers about IBD
   ___ Having a chance to talk with other patients in the registry
   ___ I will stay enrolled and answer all questionnaires no matter what

3) What would be your preferred method of filling out surveys? Rank the top 3, marking the most preferred with the number 1, the second most preferred with the number 2, and the third most preferred with the number 3.
   ___ Paper survey at home that I mail back
   ___ Paper or tablet/computer survey in the clinic
   ___ Phone survey
   ___ Email survey (secure and encrypted)
   ___ Survey on the internet/web (secure and encrypted)
   ___ Survey on a smart phone application (secure and encrypted)
   ___ Other: ____

4) At most, how often would you be willing to respond to questionnaires or surveys? Pick one answer.
   ☐ Every 1-3 months
   ☐ Every 4-6 months
   ☐ Every 7-12 months
   ☐ Every 13-24 months

5) What is the maximum length of time you would be willing to spend on surveys? Pick one answer.
   ☐ Less than 2 minutes
   ☐ 2-5 minutes
   ☐ 5-10 minutes
   ☐ 10-25 minutes

☐ >25 minutes

6) Would you consider answering surveys more often if they were shorter? For example, if the registry wanted you to report on specific symptoms, like abdominal pain, with a 1-2 minute checklist. Would you consider answering such questions, and how often? Pick one answer.
   ☐ Would not answer
   ☐ Yes, about every 1 week
   ☐ Yes, about every 2 weeks
   ☐ Yes, about every 1 month
   ☐ Yes, about every 2 months

7) If surveys are more than 10 minutes, what would be a motivating payment for you? Pick one answer.
   ☐ No payment necessary
   ☐ \$10
   ☐ \$20
   ☐ >\$30

8) What topics should the registry ask you about on surveys? Rank the top 5, marking the most important with the number 1, the second most important with the number 2, and so worth until you get to the number 5.
   ____ Medications for IBD
   ____ Medications, other than for IBD
   ____ Emotional health
   ____ Coping strategies (such as meditation or physical activity)
   ____ IBD disease activity
   ____ IBD disease complications (such as abscesses or urinary problems)
   ____ Economic effects of IBD
   ____ Diet and nutrition
   ____ Alternative therapy (such as supplements and health food preparations)
   ____ Other medical conditions
   ____ Other: ____

The following set of questions is designed to collect some information related to how you feel about giving <u>blood</u> samples to the registry.

9) At most, how often would you be willing to provide blood samples (4 tablespoons or less per sample)?
   ☐ Every 1-3 months
   ☐ Every 4-6 months
   ☐ Every 7-12 months
   ☐ Never

10) What would be a motivating payment for you to give blood samples?
   ☐ No payment necessary
   ☐ \$10
   ☐ \$20
   ☐ >\$30

The following set of questions is designed to collect some information about how you feel about giving biopsies to the registry.

11) At most, how often would you be willing to provide intestinal biopsies (during endoscopy)?
- ☐ Every time I have an endoscopy
- ☐ Every other time I have an endoscopy
- ☐ Never

12) What would be a motivating payment for you to give intestinal biopsies?
- ☐ No payment necessary
- ☐ $10
- ☐ $20
- ☐ >$30

The following set of questions is designed to collect some information about how you feel about giving stool samples to the registry.

13) At most, how often would you be willing to provide stool samples?
- ☐ Every 2 weeks
- ☐ Every 1 month
- ☐ Every 6 weeks
- ☐ Every 2 months
- ☐ Every 3 months
- ☐ Every 6 months
- ☐ Every 1 year
- ☐ Never

14) What would be a motivating payment for you to give stool samples?
- ☐ No payment necessary
- ☐ $10
- ☐ $20
- ☐ >$30

15) Would you be willing to provide stool samples between regularly scheduled clinic visits?
- ☐ Yes
- ☐ No

The following set of questions is designed to collect some information about how you feel about giving urine samples to the registry.

16) At most, how often would you be willing to provide urine samples?
- ☐ Every 2 weeks
- ☐ Every 1 month
- ☐ Every 6 weeks
- ☐ Every 2 months
- ☐ Every 3 months
- ☐ Every 6 months
- ☐ Every 1 year
- ☐ Never

17) What would be a motivating payment for you to give urine samples?
- ☐ No payment necessary
- ☐ $10
- ☐ $20
- ☐ >$30

Would you be willing to provide urine samples between regularly scheduled clinic visits?
- ☐ Yes
- ☐ No

The following set of questions is designed to find out how you have been feeling during the last two weeks. You will be asked about symptoms you are having as a result of your IBD, the way you have been feeling in general, and how your mood has been.

18) How often has the feeling of fatigue or of being tired and worn out been a problem for you during the last two weeks? Please choose one option.
- ☐ All of the time
- ☐ Most of the time
- ☐ A good bit of the time
- ☐ Some of the time
- ☐ A little of the time
- ☐ Hardly any of the time
- ☐ None of the time

19) How often during the last two weeks have you had to delay or cancel a social engagement because of your bowel problem? Please choose one option.
- ☐ All of the time
- ☐ Most of the time
- ☐ A good bit of the time
- ☐ Some of the time
- ☐ A little of the time
- ☐ Hardly any of the time
- ☐ None of the time

20) As a result of your bowel problems, how much difficulty have you had doing leisure or sports activities over the last two weeks? Please choose one option.
- ☐ A great deal of difficulty, activities made impossible
- ☐ A lot of difficulty
- ☐ A fair bit of difficulty
- ☐ Some difficulty
- ☐ A little difficulty
- ☐ Hardly any difficulty
- ☐ No difficulty; the bowel problems did not limit sports or leisure activities

21) How often during the last two weeks have you been troubled by pain in the abdomen? Please choose one option.
- ☐ All of the time
- ☐ Most of the time

- ☐ A good bit of the time
- ☐ Some of the time
- ☐ A little of the time
- ☐ Hardly any of the time
- ☐ None of the time

22) How often during the last two weeks have you felt depressed or discouraged? Please choose one option.
    - ☐ All of the time
    - ☐ Most of the time
    - ☐ A good bit of the time
    - ☐ Some of the time
    - ☐ A little of the time
    - ☐ Hardly any of the time
    - ☐ None of the time

23) Overall, in the last two weeks, how much of a problem have you had passing gas? Please choose one option.
    - ☐ A major problem
    - ☐ A big problem
    - ☐ A significant problem
    - ☐ Some trouble
    - ☐ A little trouble
    - ☐ Hardly any trouble
    - ☐ No trouble

24) Overall, in the last two weeks, how much of a problem have you had maintaining or getting to the weight you would like to be? Please choose one option.
    - ☐ A major problem
    - ☐ A big problem
    - ☐ A significant problem
    - ☐ Some trouble
    - ☐ A little trouble
    - ☐ Hardly any trouble
    - ☐ No trouble

25) How often during the last two weeks have you felt emotionally relaxed and free of tension? Please choose one option.
    - ☐ All of the time
    - ☐ Most of the time
    - ☐ A good bit of the time
    - ☐ Some of the time
    - ☐ A little of the time
    - ☐ Hardly any of the time
    - ☐ None of the time

26) How much of the time during the last two weeks have you been troubled by the feelings of having to go to the toilet even though your bowels were empty? Please choose one option.
    - ☐ All of the time

- ☐ Most of the time
- ☐ A good bit of the time
- ☐ Some of the time
- ☐ A little of the time
- ☐ Hardly any of the time
- ☐ None of the time

27) How much of the time during the last two weeks have you felt angry as a result of your bowel problems? Please choose one option.
- ☐ All of the time
- ☐ Most of the time
- ☐ A good bit of the time
- ☐ Some of the time
- ☐ A little of the time
- ☐ Hardly any of the time
- ☐ None of the time

The following set of questions is designed to collect some demographic information about you.

28) What is your age? Enter in years.

years

29) What is your gender?

☐ Male ☐ Female ☐ Prefer not to answer

30) Please indicate how you describe your race and ethnicity. Select all that apply.
- ☐ White, non-Hispanic
- ☐ White, Hispanic
- ☐ Black, non-Hispanic
- ☐ Black, Hispanic
- ☐ Asian
- ☐ Pacific Islander
- ☐ Native American
- ☐ Other:
- ☐ Prefer not to answer

31) Please indicate the highest level of formal education you have completed.
- ☐ Grade School
- ☐ Some High School
- ☐ High School
- ☐ Some College
- ☐ College
- ☐ Post-graduate
- ☐ Prefer not to answer
