# Supplementary material for: Clinical patient registry recruitment and retention: a survey of patients in two chronic disease registries
Source: BMC Med Res Methodol. 2017 Apr 17;17:59. doi: 10.1186/s12874-017-0343-3 (PMC5392954; doi:10.1186/s12874-017-0343-3)
Supplement: Supplementary file 2 — This table describes the preferred methods of completing survey by gender after combining both cohorts. (DOCX 11 kb) [file 12874_2017_343_MOESM2_ESM.docx]

**Table S1: Preferred methods of completing survey by gender after combining both cohorts**

|  | Male | Female | p-value |
| --- | --- | --- | --- |
| Paper surveys mailed home | 0.7 ± 1 | 1.3 ± 1.3 | 0.002 |
| Paper or tablet at clinic | 0.5 ± 0.8 | 0.8 ± 1.1 | 0.006 |
| Phone survey | 0.1 ± 0.5 | 0.1 ± 0.4 | 0.83 |
| E-mail survey | 1.6 ± 1.2 | 1.7 ± 1.2 | 0.69 |
| Internet survey | 1.9 ± 1.3 | 1.1 ± 1.2 | <.0001 |
| Survey using a PDA (application) | 0.7 ± 1 | 0.5 ± 0.8 | 0.02 |

* To allow for easier comparison across items and across registries, we created a score from 0-3, where 0 signified no ratings of an item in the top 3, and 3 signified the top rated item across all respondents. The ratings were 3 = most important, 2 = second most important, and 1 = third most important. For items not rated in the top 3, zero was assigned. P-values calculated using Kruskal-Wallis tests.
